# Supplementary material for: Phenotypic Characterization by Single-Cell Mass Cytometry of Human Intrahepatic and Peripheral NK Cells in Patients with Hepatocellular Carcinoma
Source: Cells. 2021 Jun 14;10(6):1495. doi: 10.3390/cells10061495 (PMC8231799; doi:10.3390/cells10061495)
Supplement: Supplementary file 1 [file cells-10-01495-s001.zip › cells-1211382-supplementary.pdf]

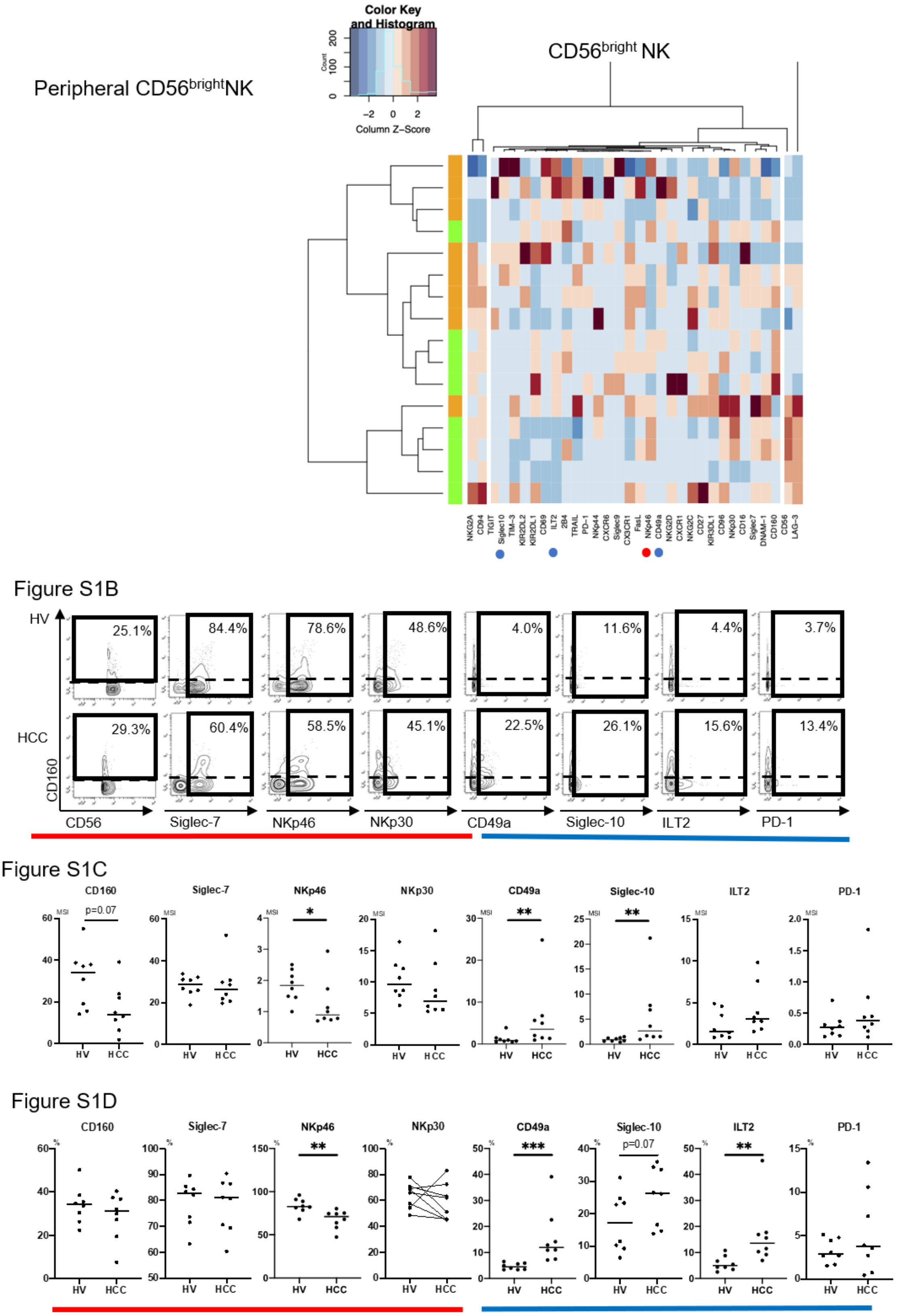

Figure S1. Phenotypic characterization of peripheral CD56brightNK cells from HCC patients. (**A**) Heatmap for hierarchical cluster analysis. The horizontal axis represents 32 surface markers on CD56brightNK cells. The vertical axis represents samples of HCC patients and healthy volunteers (HVs). The heat map indicates the deviation score of expression levels of surface markers in any sample. The blue region represents the downregulated markers and the red region represents the upregulated markers. Red circles indicate the surface markers which were expressed highly on CD56dimNK cells from HVs than those from HCC patients (NKp46). Blue circles indicate the surface markers which were expressed highly CD56dimNK cells from HCC patients than those from HVs (CD49a, Sigle-10 and ILT2). (**B**) Representative mass cytometry plots, (**C**)

quantification (MSI) and (**D**) percentages (%)of CD160, Siglec-7, NKp46, NKp30, CD49a, Siglec-10, ILT2 and PD-1 expressions on CD56^{bright}NK cells from HCC patients (n=8) and HVs (n=8). Data are presented as individual values with a mean line. *, p< 0.05; **, p< 0.01; ***, p < 0.001; ****, p < 0.0001 by the Mann–Whitney U-test. Red and blue lines indicate activating and inhibitory receptors.

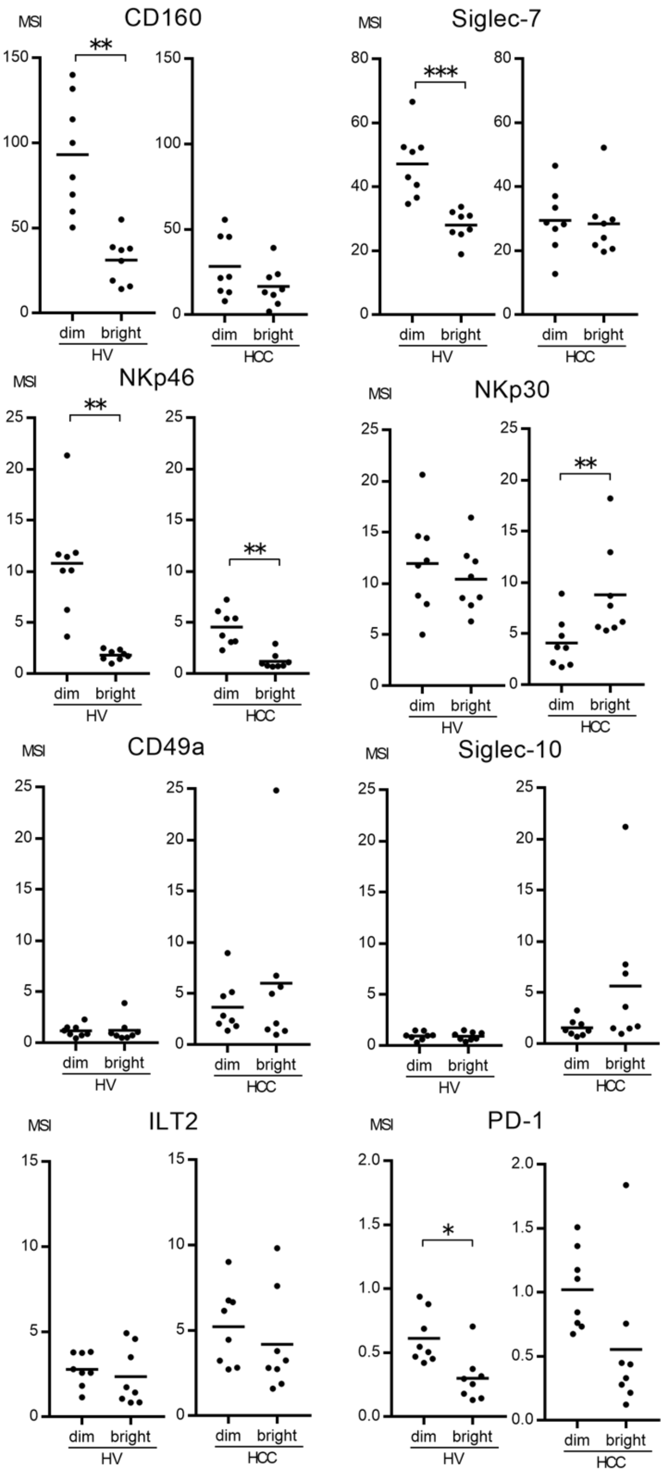

Figure S2. Comparison of CD160, Siglec-7, NKp46, NKp30, CD49a, Siglec-10, ILT2 and PD-1 expression levels between peripheral CD56^{dim} and CD56^{bright}NK cells in HVs and HCC patients. Quantification of CD160, Siglec-7, NKp46, NKp30, CD49a, Siglec-10, ILT2 and PD-1 expressions (MSI) on peripheral CD56^{dim} and CD56^{bright}NK cells. *, p< 0.05; **, p< 0.01; ***, p< 0.001 by Wilcoxon signed-rank test. dim, CD56^{dim}NK cells; bright, CD56^{bright}NK cells; HV, healthy volunteer; HCC, patients with hepatocellular carcinoma.

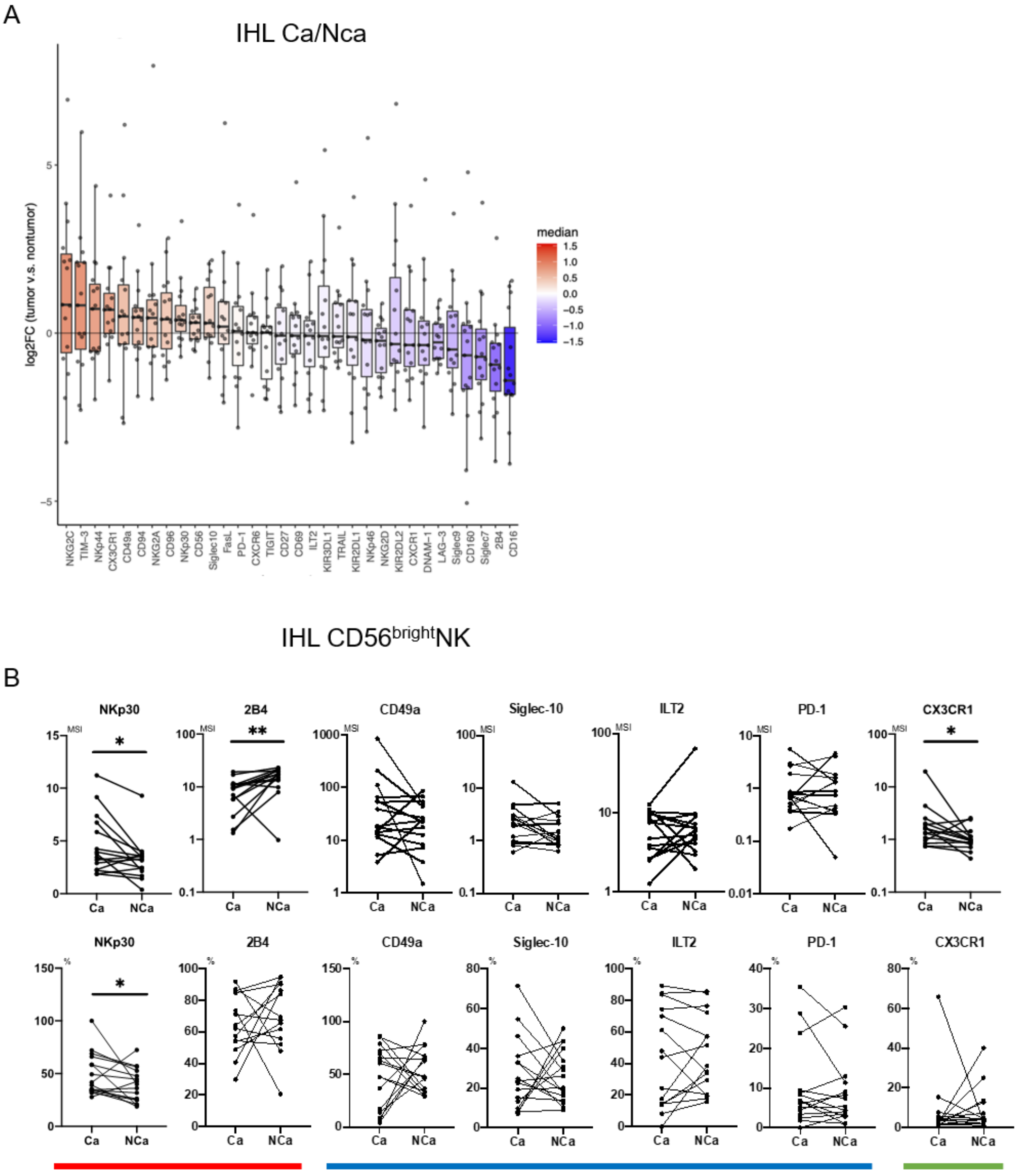

Figure S3. Phenotypic characterization of intrahepatic CD56brightNK cells from HCC patients. (A) The relative expression of the surface markers on Ca-CD56brightNK cells and NCa-CD56brightNK cells (n=16). The horizontal axis indicates log fold changes of MSI in Ca-CD56brightNK cells with respect to in NCa-CD56brightNK cells. All boxplots show median, 1.5 Inter-Quartile Range (IQR) and upper and lower quantile. (B) Quantification (MSI) and frequency (%) of NKp30, 2B4, CD49a, Siglec-10, ILT2, PD-1 and CX3CR1 expressions on Ca-CD56brightNK cells and NCa-CD56brightNK cells. *, $p < 0.05$; **, $p < 0.01$ by paired t-test. IHL, intrahepatic lymphocyte; Ca, HCC tissues; NCa, noncancerous adjacent liver tissues. Red, blue, green lines indicate activating, inhibitory and chemokine receptors, respectively.

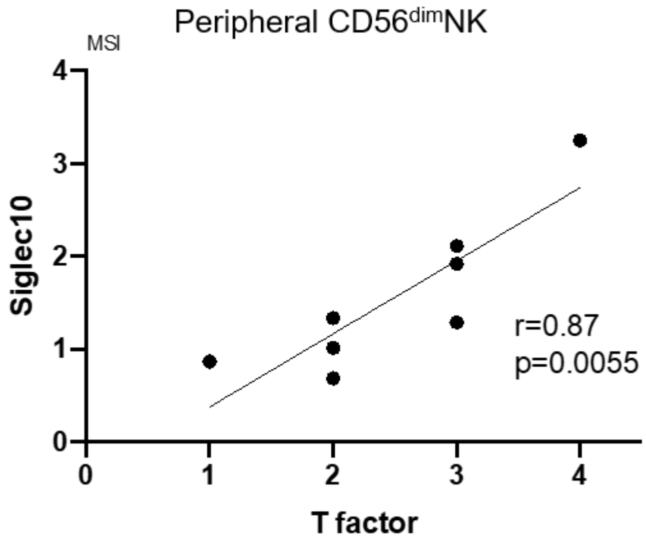

Figure S4. Correlation between Siglec-10 expression on CD56^{dim}NK cells and T factor in HCC patients. P values and correlation coefficients (r) were calculated with Spearman's correlation test. MSI, median signal intensity. The Liver Cancer Study Group of Japan (LCSGJ) staging classification: Criteria 1. Solitary tumor. 2. Diameter < 2 cm. 3. No vascular or bile duct invasion. T1, All 3 criteria are fulfilled; T2, Two of the 3 criteria are fulfilled; T3, one of the 3 criteria are fulfilled; T4, None of the 3 criteria are fulfilled or ruptured HCC.

Table S1. Clinicopathological characteristics of HCC patients for the analysis of the frequency of immune cells.

	HV	HCC
Gender (male/ female)	8 / 0	19 / 20
Age (years)	31.4 ± 6.7	80.4 ± 8.3
Etiology		HBV 2 / HCV 24 / NAFLD 10 / other 3
Child pugh score		6.1 ± 1.1
Child pugh classification (A / B / C)		26 / 13 / 0
Ascites +/-		8 / 31
Varix +/-		4 / 35
T factor (1/2/3/4)		3 / 14 / 18 / 4
N factor (0/1)		38 / 1
M factor (0/1)		38 / 1
Stage (I/II/IIIa/IIIb/IV)		3 / 14 / 18 / 3 / 1
Hb (g/dl)	14.5 ± 1.0	11.5 ± 2.0
Platelet (×10^4/μl)	25.5 ± 5.6	11.8 ± 5.8
AST (IU/l)	22 ± 6	98 ± 102
ALT (IU/l)	21 ± 8	61 ± 73
gGTP (IU/l)	23 ± 7	117 ± 187
ChE (U/L)	N.T.	173 ± 69
T-Bil (mg/dl)	0.6 ± 0.1	1.1 ± 0.5
Alb (g/dl)	4.2 ± 0.3	3.2 ± 0.5
PT (%)	N.T.	91 ± 20
Cre (mg/dl)	0.85 ± 0.08	0.86 ± 0.31
T-chol (mg/dl)	N.T.	169 ± 39
AFP (ng/ml)	N.T.	7554 ± 31953
DCP (mAU/ml)	N.T.	3998 ± 7797
Fib-4 index	N.T.	9.8 ± 6.4
NH3 (μg/dL)	N.T.	52 ± 40

Hb, hemoglobin; AST, aspartate aminotransferase; ALT, alanine aminotransferase; gGTP, γ-Glutamyl TransPeptidase; ChE, cholinesterase; T-Bil, Total bilirubin; Alb, Albumin; AFP, α-fetoprotein; DCP, Des-γ-carboxy prothrombin; Fib-4, Fibrosis-4; HBV, hepatitis B virus; HCV, hepatitis C virus; NAFLD, nonalcoholic fatty liver disease; N.T., not tested.

Table S2. Clinicopathological characteristics of HCC patients for the analysis of surface markers on NK cells.

	HCC
Gender (male/ female)	3 / 5
Age (years)	80.5 ± 13.1
Etiology	HBV 2 / HCV 4 / NAFLD 2
Child-Pugh score	6.1 ± 1.1
Child-Pugh classification (A / B / C)	5 / 3 / 0
Ascites +/-	3 / 5
Varix +/-	2 / 6
T factor (1/2/3/4)	1 / 3 / 3 / 1
N factor (0/1)	8 / 0
M factor (0/1)	8 / 0
Stage (I/II/IIIa/IIIb/IV)	1 / 3 / 3 / 1 / 0
Hb (g/dl)	11.4 ± 1.2
Platelet ($\times 10^4$/μl)	12.0 ± 7.3
AST (IU/l)	115 ± 64
ALT (IU/l)	50 ± 27
gGTP (IU/l)	76 ± 61
ChE (U/L)	146 ± 48
T-Bil (mg/dl)	1.1 ± 0.5
Alb (g/dl)	3.3 ± 0.5
PT (%)	89 ± 19
Cre (mg/dl)	0.74 ± 0.21
T-chol (mg/dl)	158 ± 39
AFP (ng/ml)	24512 ± 64739
DCP (mAU/ml)	3412 ± 6283
Fib-4 index	11.2 ± 5.3
NH3 (μg/dL)	52 ± 32

Hb, hemoglobin; AST, aspartate aminotransferase; ALT, alanine aminotransferase; gGTP, γ-Glutamyl TransPeptidase; ChE, cholinesterase; T-Bil, Total bilirubin; Alb, Albumin; AFP, α-fetoprotein; DCP, Des-γ-carboxy prothrombin; Fib-4, Fibrosis-4; HBV, hepatitis B virus; HCV, hepatitis C virus; NAFLD, nonalcoholic fatty liver disease.

Table S3. Clinicopathological characteristics of patients for the analysis of intrahepatic NK cells

						Laboratory data							Tumor / Liver Characterrisitics				
Case	Age	Gender	Etiology	CH or LC	PBMC	Plt x10^4 (/ul)	Alb (g/dl)	PT (%)	PIVKA-II	AFP	F stage	T factor	Differe-ntiation	Size (cm)	St / Mt	Outcome	Observation period (month)
1	81	M	NASH	LC	○	14.8	3.5	121.6	419	3.9	4	3	mod	3.6	St	Dead	5
2	64	M	C	CH	○	8.4	4	128.3	302	30.8	3	4	mod	7	Mt	Alive	8
3	87	F	NASH	LC	○	18.5	4.1	110.7	1793	6.4	4	2	mod	6.5	St	Alive	12
4	84	M	NASH	CH	○	22.6	3.7	130	332	2.4	2	1	well	1	St	Alive	14
5	77	M	NASH	CH	○	19.3	4.4	130	51	4.9	2	2	poorly	7	St	Alive	3
6	59	M	C	CH	○	8.2	3.5	82	50000	200.8	3	2	mod	8	St	Rec	18
7	81	M	NASH	CH	○	22.2	4.4	96	2959	6.7	2	2	mod	6.5	St	Alive	19
8	69	M	Alc	CH	○	15.5	4.4	100	866	48.7	N.T.	2	mod	5	St	Rec	25
9	77	F	C	CH	N.T.	10.6	4.1	100	23	45.8	3	1	poorly	1.5	St	Alive	33
10	70	F	C	LC	N.T.	10	3.7	91	67	63.4	4	3	mod	6.7	St	Alive	31
11	74	M	Alc	CH	N.T.	21.1	4	100	118	3.4	N.T.	2	mod	7.8	St	Alive	30
12	85	M	NASH	CH	N.T.	16.2	4.5	95	765	91.4	N.T.	2	mod	4.3	St	Alive	30
13	45	M	B	LC	N.T.	14.2	4.2	100	1200	28.6	4	2	mod	1	Mt	Alive	29
14	65	M	Alc	LC	N.T.	12	4.3	92	N.T.	N.T.	4	1	mod	1.5	St	Alive	28
15	45	M	B	LC	N.T.	16.8	4.7	84	371	6.8	4	4	mod	3.5	Mt	Alive	27
16	58	M	C	CH	N.T.	14.5	4.5	90	4182	3586.5	3	3	mod	3	St	Dead	26

PBMC, Peripheral Blood Mononuclear Cells; Plt, platelet; Alb, Alubmin; PT, prothrombin time; PIVKA-Ⅱ, protein induced by vitamin K absence or antagonist Ⅱ; AFP, α-fetoprotein; F stage, stage of liver fibrosis; T factor, Tumor factor in The

Liver Cancer Study Group of Japan (LCSGJ) tumor-node-metastasis (TNM) staging for hepatocellular carcinoma; St, Single tumor; Mt, multiple tumors; NASH, nonalcoholic steatohepatitis; C, hepatitis C virus; B, hepatitis B virus; Alc, Alcoholic hepatitis; CH, chronic hepatitis; LC, liver cirrhosis; N.T., not tested; mod, moderate; Rec, Recurrence.

Table S4. Antibodies used for mass cytometry in Figure 1.

Channel	Isotope	Marker	Function of Markers	Clone	Staining Method	PC/IH
89	Y	CD45	lymphocytes identification	HI30	surface	PC
142	Nd	CD19	B cell identification	HIB19	surface	PC
143	Nd	HLA-DR	DC identification	L243	surface	PC
145	Nd	CD4	CD4 T identification	PRA-T4	surface	PC
147	Sm	CD11c	mDC identification	Bu15	surface	PC
151	Eu	CD123 (IL-3R)	pDC identification	6H6	surface	PC
154	Sm	TCR Va7.2	MAIT identification	3C10	surface	PC
155	Gd	CD56 (NCAM)	NK identification	B159	surface	PC
160	Gd	CD14	monocytes identification	M5E2	surface	PC
164	Dy	CD161	MAIT inhibitory	HP-3G10	surface	PC
167	Er	CD3	T cell inhibitory	UCHT1	surface	PC
168	Er	CD8a	CD8a T identification	SK1	surface	PC
170	Er	TCRVa24-Ja18	iNKT cell identification	6b11	surface	PC
171	Yb	CD185 (CXCR5)	Tfh cell identification	RF8B2	surface	PC
172	Yb	CD45RA	Naïve T identification	HI100	surface	PC
173	Yb	TCR VDelta2	γδ T identification	B6	surface	IH
209	Bi	CD16	cytotoxicity NK identification	3G8	surface	PC

HLA-DR, human leukocyte antigen-DR; TCR, T cell receptor; Tfh, follicular helper T; IH, in-house (labeling kit); PC, pre-conjugated

Table S5. Antibodies used for mass cytometry for the analysis of surface markers on NK cells in Figure 2, Figure 3, and Figure 4.

Channel	Isotope	Marker	Function of Markers	Clone	Staining Method	PC/IH
89	Y	CD45	lymphocytes identification	HI30	surface	PC
106	Cd	CD3	T cell identification	UCHT1	surface	IH
112	Cd	CD85j (ILT2)	NK inhibition	292305	surface	IH
113	Cd	CD314 (NKG2D)	NK activation	149810	surface	IH
114	Cd	CD56 (NCAM)	NK identification	HCD56	surface	IH
116	Cd	TIGIT	inhibitory immune checkpoint	741182	surface	IH
141	Pr	CD27	NK differentiation	O323	surface	IH
144	Nd	CD69	NK activation	FN50	surface	PC
145	Nd	KIR2DL1	NK inhibition	MAB1844	surface	IH
146	Nd	CXCR6	NK differentiation	56811	surface	IH
147	Sm	CD159c (NKG2C)	NK activation	MAB1381	surface	IH
148	Nd	Siglec-10	Siglec family	AF2130	surface	IH
149	Sm	Siglec-9	Siglec family	K8	surface	IH
150	Nd	CXCR1	NK activation	42705	surface	IH
151	Eu	CD96	NK activation	MAB6199	surface	IH
152	Sm	CD328 (Siglec-7)	Siglec family	194211	surface	PC
153	Eu	TIM-3	inhibitory immune checkpoint	F38-2E2	surface	PC
154	Sm	CD49a	NK differentiation	AF5676	surface	PC
155	Gd	CX3CR1	NK differentiation	AF5825	surface	IH
156	Gd	CD94	NK inhibition	131412	surface	IH
159	Tb	CD337 (NKp30)	NK activation	Z25	surface	PC
160	Gd	CD14	monocytes identification	M5E2	surface	PC
162	Dy	CD335 (NKp46)	NK activation	BAB281	surface	IH
164	Dy	TRAIL	NK activation	RIK-2	surface	IH
165	Ho	CD223 (LAG-3)	inhibitory immune checkpoint	11C3C65	surface	PC
167	Er	CD158e1 (KIR3DL1)	NK inhibition	DX9	surface	PC
168	Er	CD160	NK activation	688327	surface	IH
169	Tm	CD159a (NKG2A)	NK inhibition	Z199	surface	PC
170	Er	CD244 (2B4)	NK activation	MAB10393	surface	IH
171	Yb	CD226 (DNAM-1)	NK activation	DX11	surface	PC
172	Yb	FasL	NK activation	NOK-1	surface	IH
173	Yb	CD158b (KIR2DL2/L3)	NK inhibition	DX27	surface	PC
174	Yb	CD279 (PD-1)	inhibitory immune checkpoint	EH12.2H7	surface	PC
175	Lu	CD336 (NKp44)	NK activation	MAB22491	surface	IH
209	Bi	CD16	cytotoxicity NK identification	3G8	surface	PC

ILT2, Ig-like transcript 2; human leukocyte antigen-DR; TIGIT, T-cell immunoreceptor with immunoglobulin and ITIM domains; KIR, Killer cell Immunoglobulin-like Receptor; Siglec, sialic acid binding immunoglobulin-like lectin; TIM-3, T-cell immunoglobulin and mucin domain 3; TRAIL, tumor necrosis factor- related apoptosis-inducing ligand; LAG-3, Lymphocyte activation gene 3; DNAM-1, DNAX Accessory Molecule-1; PD-1, programmed cell death-1.
